# Modeling, Fabrication and Integration of Wearable Smart Sensors in a Monitoring Platform for Diabetic Patients

**DOI:** 10.3390/s21051847

**Published:** 2021-03-06

**Authors:** Chiara De Pascali, Luca Francioso, Lucia Giampetruzzi, Gabriele Rescio, Maria Assunta Signore, Alessandro Leone, Pietro Siciliano

**Affiliations:** National Research Council of Italy, Institute for Microelectronics and Microsystems (CNR-IMM), 95121 Lecce, Italy; chiara.depascali@le.imm.cnr.it (C.D.P.); gabriele.rescio@cnr.it (G.R.); mariaassunta.signore@cnr.it (M.A.S.); alessandro.leone@cnr.it (A.L.); pietroaleardo.siciliano@cnr.it (P.S.)

**Keywords:** diabetic foot ulcer, finite element method simulations, bioheat transfer, smart multisensory platform, AlN piezoelectric pressure sensor, glucose sensor, microfabrication techniques

## Abstract

The monitoring of some parameters, such as pressure loads, temperature, and glucose level in sweat on the plantar surface, is one of the most promising approaches for evaluating the health state of the diabetic foot and for preventing the onset of inflammatory events later degenerating in ulcerative lesions. This work presents the results of sensors microfabrication, experimental characterization and FEA-based thermal analysis of a 3D foot-insole model, aimed to advance in the development of a fully custom smart multisensory hardware–software monitoring platform for the diabetic foot. In this system, the simultaneous detection of temperature-, pressure- and sweat-based glucose level by means of full custom microfabricated sensors distributed on eight reading points of a smart insole will be possible, and the unit for data acquisition and wireless transmission will be fully integrated into the platform. Finite element analysis simulations, based on an accurate bioheat transfer model of the metabolic response of the foot tissue, demonstrated that subcutaneous inflamed lesions located up to the muscle layer, and ischemic damage located not below the reticular/fat layer, can be successfully detected. The microfabrication processes and preliminary results of functional characterization of flexible piezoelectric pressure sensors and glucose sensors are presented. Full custom pressure sensors generate an electric charge in the range 0–20 pC, proportional to the applied load in the range 0–4 N, with a figure of merit of 4.7 ± 1 GPa. The disposable glucose sensors exhibit a 0–6 mM (0–108 mg/dL) glucose concentration optimized linear response (for sweat-sensing), with a LOD of 3.27 µM (0.058 mg/dL) and a sensitivity of 21 µA/mM cm^2^ in the PBS solution. The technical prerequisites and experimental sensing performances were assessed, as preliminary step before future integration into a second prototype, based on a full custom smart insole with enhanced sensing functionalities.

## 1. Introduction

The problematic of diabetic foot comprises the onset of infection, ulceration and/or destruction of deep tissues aggravated by neurological pathology and/or peripheral vascular disease in the lower limbs of patients affected by diabetes [1]. Neuropathy, ischemia and infection represent the main pathological components that lead to diabetic foot complications. Neuropathy and ischemia are initiating factors, and most often, they coexist together as neuro-ischemia, whereas infection is mostly a consequent state. Neuropathy can hinder the motor capability, with problems ranging from muscle weakness up to paresis. It can cause a sensory deficit, with loss of sensation of heat, pain and pressure, triggering autonomic dysfunctions, which induce anomalous local vasodilatation and reduced sweating, with consequent loss of skin integrity and tendency to develop skin fissuring and infections [2,3]. Ischemia is a particularly serious form of peripheral arterial disease, which determines a marked reduction of blood flow toward the lower extremities due to a severe blockage in the arteries of limbs. In diabetic patients, ischemia represents a troubling state in presence of foot infections and/or ulceration, being the blood supply inadequate to meet the increased metabolic demand of the lesion underway. This because a severe complication of the poor blood circulation is the onset of hard-to-heal wounds and sores in the lower extremities, which, if not treated early or left untreated, can entail spreading infection, ulceration and gangrene of ulcer, increasing the risk of amputation of the affected part of the lower limb [4]. While neuropathic wounds have a good chance of healing within five to six months, ischemic or neuro-ischemic injuries have a longer and more difficult progress because the blood perfusion is inadequate with respect to the demand for blood from the damaged tissue [5].

In the last years, numerous studies have demonstrated that plantar pressure is higher, compared to standard values, in people with diabetic peripheral neuropathy and/or neuro-ischemia, and in people with a history of diabetic foot ulcers [6,7,8,9,10,11,12]. As consequence, this plantar overpressure leads to chronic foot lesions in diabetic patients [13,14]. The monitoring of plantar pressure is crucial for assessing the status of health of diabetic feet because repetitive mechanical stress [15], combined with loss of protective sensation on the plantar surface [16], is considered the initiating cause for the onset of the above-mentioned main skin pathologies [17]. To avoid foot ulcerations, an accurate regular feet examination from physical/structural abnormalities (like calluses, hammertoe, abnormal bony structures, or other aberrations to typical anatomy) should be performed by evaluating the pressure on plantar tissue, both on bare feet and in footwear. The most used methods for the measurements of plantar pressure are the following: (a) pressure platforms [18], which provide a direct quantitative measure of plantar pressure; (b) force platforms combined with visualization techniques [19], which overcome the limitation of simple platform allowing to measure also the deformation force on the foot; (c) ex vivo analysis conducted on tissue samples to evaluate the stress endured by the tissue below the skin surface [20]; and, finally, (d) finite element analysis [21], which models and simulates the foot tissue for evaluating its mechanical behavior (stress-strain relationship) under different loading conditions.

Today, footwear and off-loading devices, i.e., removable casts and specifically made shoes, are employed to mitigate excessive plantar pressures, helping diabetic foot ulcer treatment [22]. However, these devices generally intervene only when the ulcer has already developed [23]. Therefore, the challenge in this field is to predict ulcers formation to prevent its development. Thus, the best strategy in prevention and care of diabetic foot pathology is the quantitative evaluation of plantar pressure and data recording to (i) identify sites with high risk of wound formation, predicting potential ulcer occurrence, and/or (ii) guide effectively an adequate pressure relief from ulcerated sites to promote wound healing. The continuous in-shoe pressure analysis by embedded low-cost pressure sensors could remove the need for directed walking over pressure platform systems. Sensors-equipped insoles are very attractive solution, which allows the patients for wearing them outside of the clinic for continuous data logging analysis [24]. Commercial pressure sensors, mainly force sensitive resistors (FSR), have been used as sensing element and embedded into insole, until recently [25,26,27,28,29]. Despite the attempts made to achieve an effective integration of commercial pressure sensors into insole, they have revealed some limitations in terms of accuracy, precision, level of integration, cost, wearability and comfort [30,31]. Microfabrication techniques represent an effective tool to realize suitable pressure sensors to integrate into insole, by respecting nodal requirements such as flexibility for a better embodiment, small size to avoid any hindrance to normal feet movements, specific geometry to not inflict damage to the feet, suitability for low power operation, measurability of pressure in the range 0–1000 kPa [32]. Some examples of microfabricated pressure sensors for diabetic plantar pressure measurement can be found in the literature [33,34].

Besides plantar pressure, plantar temperature is another important indicator of health conditions of diabetic foot [35]. Inflammation in the underlying tissues and changes in blood flow can affect skin temperature, and for this reason they are considered the two main factors able to give an alert about the early onset of possible tissue abnormalities [36]. As for high pressure, high temperature on the plantar is also an alerting factor, which, when associated to reduced or complete loss of sensation, can predispose the patient to risk of foot injury/ulceration. In the recent past decades, numerous studies demonstrated that some complications correlated to diabetic disease induce an anomalous variation of the foot plantar temperature, and the monitoring of feet temperature in diabetic patients can help for earlier recognition and treatment of diabetic foot problems [37]. One of the most interesting methods for measuring the foot temperature rely on thermographic inspection and analysis of thermal images of at-risk diabetic feet. In the last years, thanks to the development of even more sophisticated systems of acquisition and processing of thermal images, thermography in medicine has experienced a renewed interest, becoming an alternative to conventional medical thermometers for monitoring body temperature.

Infrared thermal imaging technique was exploited to analyze the temperature distribution on plantar foot of 112 subjects with type 2 diabetes [38]. The study demonstrated that patients with diabetic neuropathy tend to have a higher foot temperature (32–35 °C) compared to patients without neuropathy (27–30 °C). In absence of traumatic wounds, it was observed an increase of temperature at sites with high risk of ulceration, compared with the opposite foot, which can be plausibly associated with a localized inflammation and enzymatic autolysis of tissue [39]. As regards the sites that are likely to ulcerate, a study about a population of 87 patients with active foot ulcer [40], calculated the probability of occurrence of ulcerative lesion under the metatarsal heads (56.3%), toes (32.1%), and heel (1.9%). Numerous works correlated the variations of plantar foot temperature to neuropathy and peripheral vascular diseases [41,42,43,44].

Another important aspect related to the health monitoring of diabetic patients is the monitoring and control of blood glucose. Diabetes is one of the most prevalent chronic diseases, causing serious damage to many of the body′s systems, especially the nerves and blood vessels because of hyperglycemia, the raised blood sugar [45]. Blood glucose levels remain above normal threshold levels, over time, when the pancreas does not produce sufficient amounts of the hormone insulin, which regulates blood glucose, or when the body cannot effectively use the insulin it produces [46]. Despite the medical recommendations, such as the daily blood glucose level monitoring and the periodic insulin shots for the continuous management of blood glucose level, several diabetic patients (about 70%) often manifest various severe diabetic complications, some of these fatal [47,48]. This is caused by an insulin overtreatment or also a negligence generated by the fear of injection-related pain for repetitive blood collection and insulin shots [49,50]. These reflections regarding the uncorrected management of glucose sensing and/or of uncontrolled drug delivery have motivated researchers, in the last two decades, for the development of novel continuous and invasive glucose monitoring systems, aimed to reduce collateral effects for improving potentially patient’s quality of life [51].

The recent non-invasive sweat-based biomarker monitoring methods using wearable biosensors have been defined as a potential solution to estimate blood glucose concentration [52,53,54]. Several sweat-based glucose sensors have been developed such as ultra-thin, flexible and stretchable enzyme-based sensors for monitoring individual health status and delivering the corresponding feedback therapy [55,56]. However, these systems still facing several challenges in terms of immobilizing enzymes, sensitivity and long-term stability. Along with the technological aspects, the correct design and protocol for glucose acquisition in sweat could also enhance the patient acceptability [54]. Here we proposed a miniaturized sensor design of flexible electrodes that allows for reliable sweat analysis. It could be integrated or connected close to the insole areas of higher temperature, where sweat production is more abundant, detecting glucose, firstly, and then able inducing sweat production, or monitoring the skin electrical conductivity and other metabolites/electrolytes as biomarkers or as controlled parameters in pre/chronic diabetic feet.

All these considerations demonstrate that long-term and continuous monitoring of relevant vital parameters on the plantar, as temperature, foot load distribution and regulating glucose levels, is crucial for prevention of diabetic foot ulcers. Several wearable technologies for the permanent monitoring of the diabetic foot were developed and discussed in literature [57,58,59]. However, the size of the devices and the combined pressure/temperature monitoring capabilities do not accommodate the requirements from either the end-users or the caregivers. As a matter of fact, just one information (pressure load or temperature map) is normally acquired. Further, the foot temperature is usually monitored on not more of five reading points is lower than five, while data acquisition and transmission is trusted to external bulky unit.

This work presents the results of sensors microfabrication, experimental characterization, and FEA based thermal analysis of a 3D foot-insole model aimed to advance in the development of a full custom smart multisensory hardware-software monitoring platform for diabetic foot. The proposed system allows for the simultaneous detection of temperature, pressure and sweat based-glucose level by means of full custom microfabricated sensors distributed on eight reading points of a smart insole. The unit for data acquisition and transmission will be fully integrated into the platform and will communicate by wireless protocol to a gateway, which is remotely accessible for a real time monitoring of foot health conditions. In addition, a finite element analysis (FEA) based bioheat transfer 3D model of insole-foot was developed using Comsol Multiphysics software, with the aim to numerically evaluate the effectiveness of the temperature sensor array to detect early signs of foot ulceration. Two different types of subcutaneous tissue lesions were modeled and the temperature distribution through the foot-insole system was evaluated as a function of the depth of the injury in the foot tissue. The microfabrication process and preliminary results of functional characterization of flexible piezoelectric pressure sensors and flexible glucose sensors are presented in this work, and the technical prerequisites for their future integration into the smart multisensory hardware-software monitoring platform are assessed. The choice of the piezoelectric transduction for the custom pressure sensor is related to its intrinsic properties which guarantee reliability, precision and a good linearity in a wide range of pressures with negligible hysteresis. Moreover, it has a very low power consumption compared to other transduction mechanisms, such as capacitive or electromagnetic.

## 2. Materials and Methods

### 2.1. Insole Architecture

**Figure 1:**
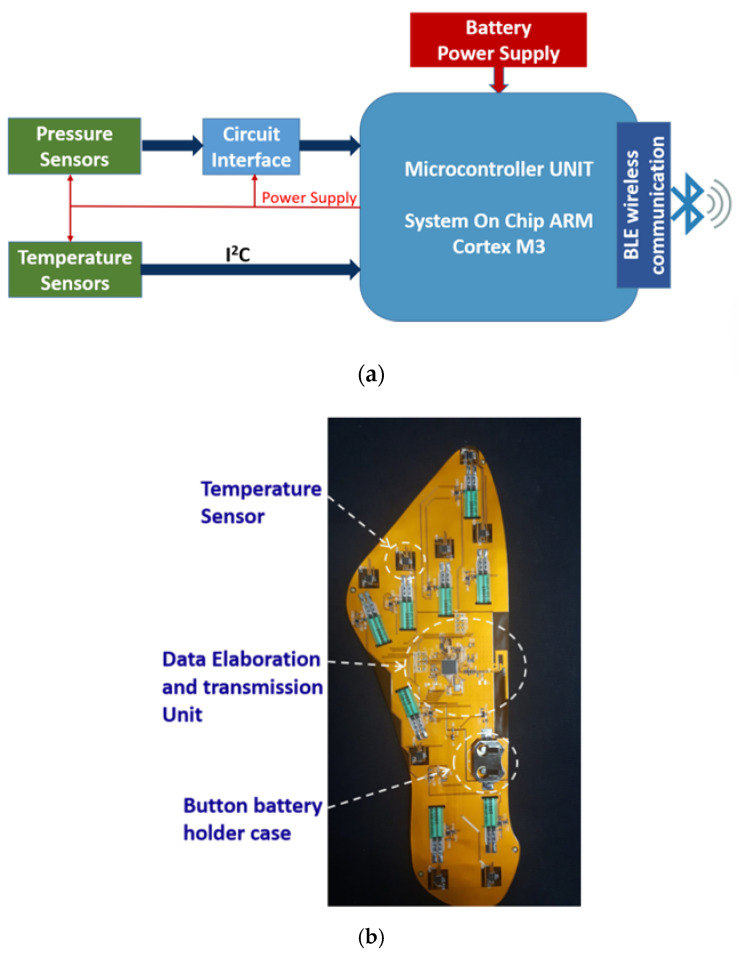
(**a**) Block diagram of the smart insole architecture; (**b**) a photo of the engineered insole.

In [60], authors presented a first prototype of smart insole for the monitoring of diabetic foot, in which commercial devices was integrated for the detection of the temperature and pressure on eight points of the plantar foot surface. Figure 1 depicts the block diagram of the implemented architecture: the sensing part is responsible for detecting data related to temperature and pressure loads on the plantar foot; the processing unit, comprising all the electronic components necessary for the acquisition and wireless transmission of data, is integrated in the insole. In particular, it includes the following:

- a 32-bit Arm Cortex-M3 multiprotocol 2.4 GHz wireless MCU, used for data acquisition, pre-processing and transmission, through a Bluetooth Low Energy (BLE) communication
- eight multiplexed channel, 12 bit Analogue to Digital converter that digitize the pressure sensors signals
- a flash memory, to avoid data loss during the transmission phase
- electronic circuits to supply the sensors (and related circuit interfaces).

The entire system is powered by a 3 V lithium-ion button battery. The maximum power consumption, in data transmission mode, is about 80 mW, and the battery life is almost 22 h in continuous transmission mode. The temperature data is acquired every 5 min, while the acquisition frequency of the pressure sensors output signal is 50 Hz.

**Figure 2:**
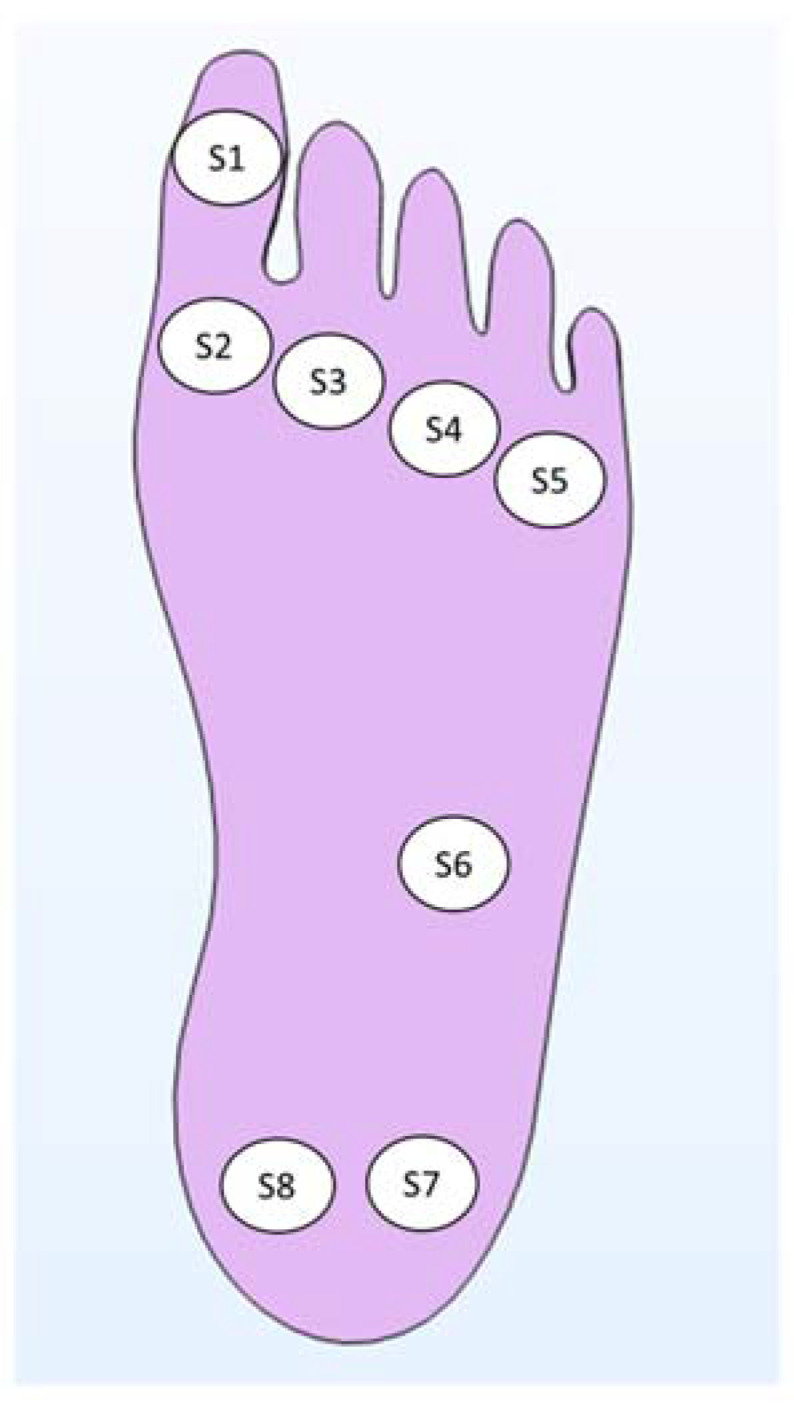
Eight sensing points distributed on the main foot points where impending subcutaneous tissue damage are likely to occur.

The smart insole was designed for achieving an accurate foot monitoring with an optimized positioning of sensors, by referring to standard insoles of european size 39 and 42 (24.7 cm and 26.7 cm of foot length, respectively). To this aim, the distribution of pressure loads was analyzed using a BTS P-Walk baropodometric platform, obtaining results in accordance with the main evidences reported in literature [61]. Figure 2 shows a schematic of the eight sensing-points distributed on the foot plantar surface.

The printed circuit board PCB integrated into the smart insole is realized on a flexible dual-layer substrate of 25 µm thick kapton. The most part of the electronic components is placed on the bottom side of the flexible board, to avoid asperities that could be potentially damaging when in direct contact with the diabetic foot. Exploiting the very thin, flat and flexible capabilities of the custom pressure and glucose sensors, it is possible and convenient to integrate them on the top side of the board, in order to guarantee a direct contact with the foot and a better pressure reading without compromising the insole comfort. The custom sweat based-glucose sensors can be integrated in the insole areas of higher temperature, where the sweat production is more abundant. As for the first prototype, the temperature sensors (Maxim MAX30205) can be used, placing them on the bottom side on the kapton substrate, with thermal sensitive pad upside; the thermal transfer from the foot to the sensing pad of the temperature sensor is maximized through a via hole realized on the board and then filled with a silver-based conductive paste.

### 2.2. Microfabricated Piezoelectric Pressure Sensors

**Figure 3:**
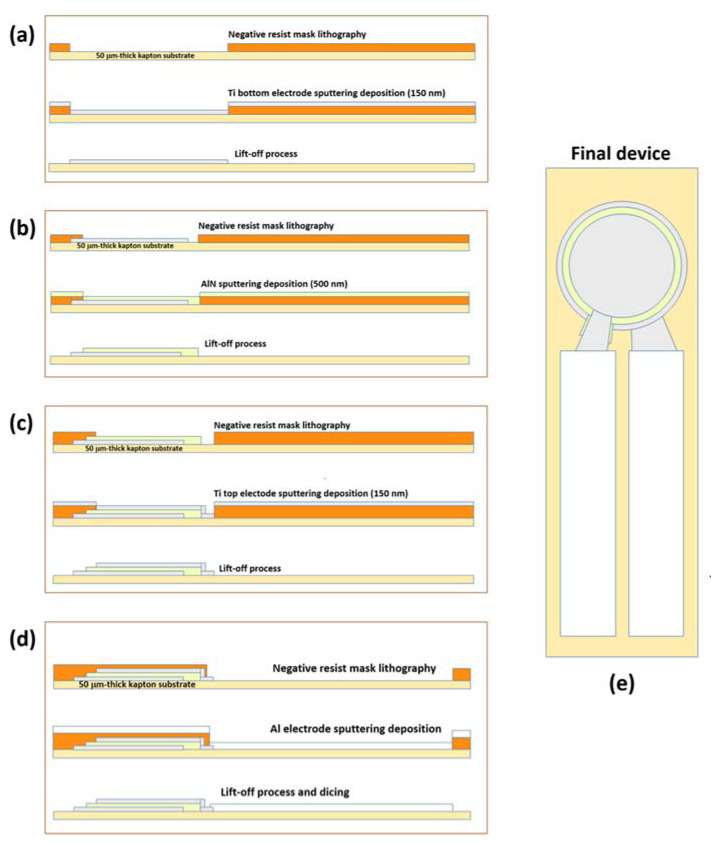
Fabrication process of piezoelectric flexible pressure sensors: (**a**) Ti bottom electrode deposition, (**b**) AlN piezoelectric layer sputtering growth, (**c**) Ti top electrode deposition, (**d**) aluminum contacts, (**e**) geometry of the final device. Adapted from [62].

The pressure sensor was realized on 50 µm-thick kapton substrate, to ensure flexibility and easy integration into the insole, avoiding negative effects on comfort. Conventional cleanroom processes of photolitography and etching were optimized to be applied on this kind of flexible substrates. The device has a concentric stacked circular structure that maximizes the charge generation, and includes a layer of piezoelectric AlN thin film sandwiched between two Ti electrodes. AlN and Ti thin films were deposited by RF magnetron sputtering, and they have thickness of 500 nm and 150 nm, respectively. The diameters of top and bottom electrodes are 3.8 mm and 4 mm, respectively. 300 nm-thick aluminum strips were deposited on Ti electrodes, as conducting pads to be linked to the reading electronic circuit and for the final packaging. Figure 3 shows the flow chart of the fabrication process of the flexible piezoelectric pressure sensor.

The dielectric constant of the AlN thin film was evaluated through C-V measurements performed in a frequency range from 20 Hz to 1 MHz. The stack Ti/AlN/Ti realized for the electrical characterization was deposited on Si/SiO_2_ substrate with a circular Ti top electrode with diameter of 200 μm. Young modulus of AlN thin film was evaluated by nanoindentation measurements by using a diamond Berkovich tip. The applied load was varied in the range 0.6 mN–4 mN, performing three measurements per each load value. Young modulus value was extracted from load and displacement relation, by using Oliver and Pharr method [63]. The sensing performance of the microfabricated pressure sensor was investigated by using an experimental custom set-up, consisting of a high precision stepper linear actuator, a 500 gr calibrated load cell, a custom designed sensor interface and a 2-channel 100 MHz oscilloscope. The sensor piezoelectric response has been evaluated by using the quasi-static method, as follows. The high precision stepper actuator, equipped with a controller, moves a rubber rod on the sensor surface at established speed, and the resulting force applied on sensor active area is evaluated by the load cell. The sensor interface amplifies and converts into a voltage signal the piezoelectric charges generated by the sensor following the mechanical stress. The oscilloscope collects and records the data. For the calibration of the set-up, the characterization procedure was first performed on a reference sample (#APC-842 from AmericanPiezoCeramics, International LTD, Mackeyville, PA, USA) [64]. Moreover, the realized interface circuit is equipped with a low noise, small size, and low power amplifier, which is a valid tool to guarantee high performance measurement in terms of accuracy.

### 2.3. Microfabricated Flexible Glucose Sensors

**Figure 4:**
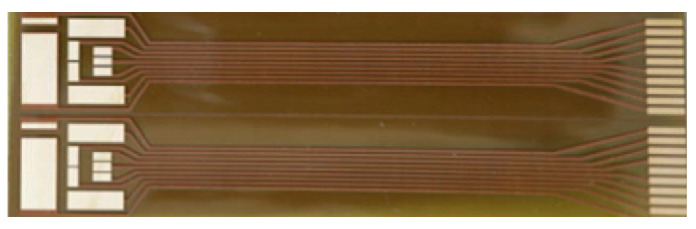
Two of the flexible designed electrodes systems dedicated to glucose measurements in sweat.

The flexible sensor proposed into present work is dedicated to glucose measurements in sweat and integrates 2 working electrodes with a common Ag/AgCl reference/counter, 2 electrodes for the sweat extraction (iontophoresis stimulus) and 2 electrodes for the future realization of a skin impedance measurement, not used in present work (Figure 4).

The devices are realized on Kapton substrate (50 µm thick) with copper tracks (35 µm thick), coated with high-purity electroplated gold (50 nm thick) in the exposed electrodes areas; the working electrode is functionalized by an enzymatic membrane of glucose oxidase (eventually lactase oxidase), while on the reference electrode a 100 µm thick layer of highly Ag-loaded silver paste (>85% wt.) was placed, subsequently chlorinated. The working electrode is made of gold, because of its less reactivity to chlorine ions present in the extracellular fluid and, in the electrode polarization range (1.1–1.5 V) with no residual current peaks observed. The chosen reference is an Ag/AgCl electrode, because of the expected stable Cl-ions concentration in the extracellular fluid and therefore the stability of the reference potential is guaranteed. The chlorination process was carried out using a current density of 0.03 mA/mm^2^, maintained between the silver electrode and an external Ag/AgCl reference electrode for 90 s (best chlorination uniformity).

#### Immobilization Protocol of Glucose Oxidase Enzyme onto the Flex Electrodes

In order to obtain a reproducible and stable system before the enzymatic membrane deposition, the substrates of electrodes were cleaned and electrochemically conditioned into a 0.1 M citrate buffer solution using cyclic voltammetry (Table 1). The immobilization step was preceded by the immersion in a 0.05 M sulfuric acid solution and conditioned using different parameters of the previous cyclic voltammetry, in order to remove all traces of any organic residues (Table 1).

The working electrodes was coated with a 6-mercaptohexanoic acid (MHA) solution and the sample was stored at 21°C for 24 h in the dark, in order to carboxylate the surface. After 24 h the substrate was rinsed with ethanol, then with de-ionized water and finally dried under N_2_, in order to remove the unreacted acid. A 2:1 mol:mol ratio of EDC:NHS was prepared and pipetted onto the array element at 28 °C for 1 h to activate the carboxylic (COOH) groups of the MHA [65]. The activated electrodes were incubated in a buffer solution that contained the glucose oxidase solution at room temperature for 2 h. After 2 h, the working electrode was washed with PBS to remove the excess of the enzymatic solution and left immersed in PBS until needed for electrochemical experimentation.

### 2.4. FEA-Based Heat Transfer Analysis of Foot-Insole

**Figure 5:**
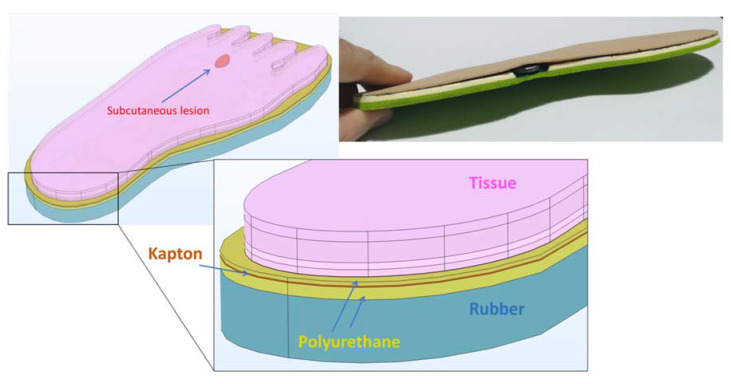
Three-dimensional model of foot-insole, with indication of the different domains simulated.

A finite element thermal analysis was performed on a 3D model of foot-insole for investigating the capability of the sensorized insole to detect early signs associated to impending foot ulcers (Figure 5). By FEA simulations, the heat transfer between foot and insole was evaluated, in presence of tissue injury located at increasing depth under the skin layer, and associated to diabetic complication (neuropathy or ischemia) from which foot ulcers are likely to occur.

In the model, the insole was defined including the following constitutive layers: 1 mm thick antibacterial polyurethane-based upper layer (in contact with the foot tissue), 0.36 mm thick flexible kapton, 0.018 mm thick copper circuit board, 3 mm thick antibacterial polyurethane-based bottom layer. For a more realistic heat transfer analysis, the thermal contribution of the shoe was also considered, including in the 3D model a rubber layer with thickness of 15 mm underneath the insole. The properties of the insole materials are reported in Table 2.

Convective heat transfer was considered at the interface between footwear and floor, with a heat transfer coefficient of 5 W/m^2^/K at environment temperature of 20 °C [66], and a mean thermal resistance of 0.02 W/mK was considered at the foot-insole interface for simulating the thermal drop trough the sock [67]. Heat transfer in the biological living tissue was modeled and solved by applying the Pennes approximation into the classical heat transfer equation, in order to consider the heat sources from blood perfusion and metabolic activity of biological tissue [68]:

(1) $$\rho C_{p}\frac{\partial T}{\partial t}+\nabla \cdot q=\rho_{b}C_{p,b}\omega_{b}\left(T_{b}-T\right)+Q_{met},$$

with:

- *ρ* is the tissue density
- *C_p_* is the specific heat capacity at constant pressure of the tissue
- *T* is the tissue absolute temperature
- ***q*** is the heat flu by conduction in the tissue
- *ρ_b_* is the blood density
- *C_p,b_* is the blood specific heat capacity at constant pressure
- *ω_b_* is the blood perfusion rate
- *T_b_* is the arterial blood temperature
- *Q_met_* is the metabolic heat source

Human foot was modeled by including the first five tissue layers: epidermis, papillary dermis, reticular dermis, fat and muscle, referring to the thermophysical properties indicated in [69,70,71,72,73,74]. The thermal analysis was performed by modeling the early stage of two types of tissue injury lesion attributable to inflammation or ischemia. Arterial blood was set to 37 °C for all the layers, except for ischemia (35 °C) [75]. The thermophysical properties of healthy and damaged tissue are reported in Table 3. The lesion was modeled with an irregular shape having height of 0.25 cm and volume of 1.5 cm^3^, and FEA simulations were performed varying the position of the lesion in the foot tissue with respect to the skin surface.

By FEA, the heat distribution from foot tissue and smart-insole was determined for each of the investigated cases, with the aim to evaluate in what conditions the array of temperature sensors (TSs) integrated on the smart insole is able to detect the presence of a subcutaneous lesion.

**Figure 6:**
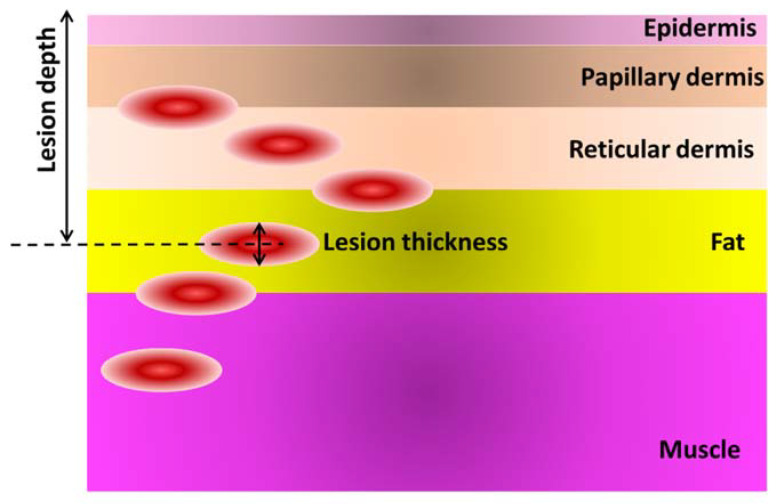
Schematic of the tissue multilayer, with indication of the six investigated depth of lesion (inflammation or ischemia) in the foot tissue.

Six cases of inflamed or ischemic subcutaneous injury were considered, with center of the lesion located at (i) 1.6 mm, (ii) 2.6 mm, (iii) 3.4 mm, (iv) 5.9 mm, (v) 8.4 mm and (vi) 9.7 mm, from the skin surface (Figure 6).

## 3. Results and Discussion

### 3.1. FEA Simulation Results

**Figure 7:**
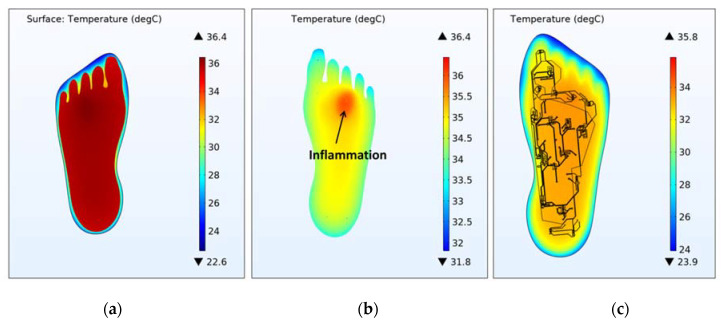
3D temperature distribution on the foot-insole model, for 2.6 mm deep inflammation nearby S4 sensor. (**a**) Top view of the 3D foot-insole model. Plane view of: (**b**) foot plantar; (**c**) engineered smart insole Kapton substrate.

**Figure 8:**
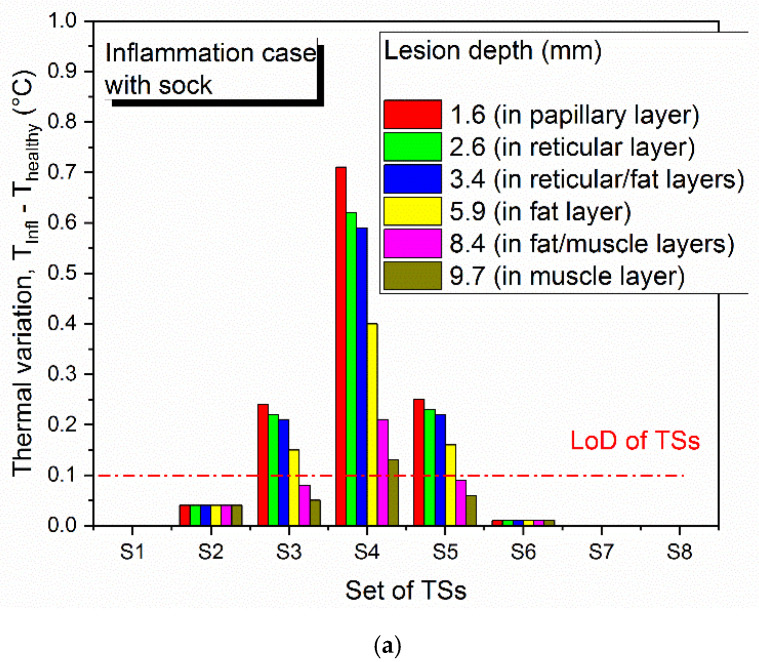

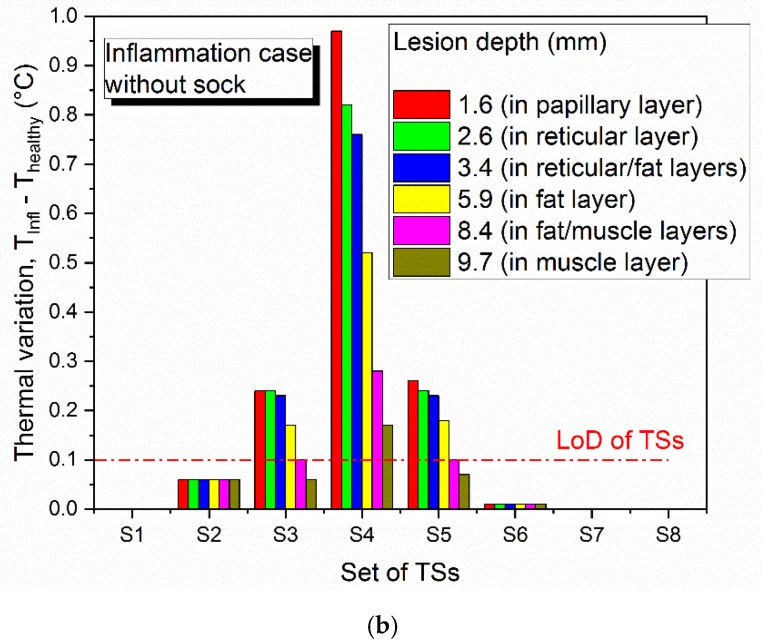
Limit of detection of thermal sensors (TSs) for subcutaneous inflamed lesion located in proximity of S4, (**a**) with sock and (**b**) without sock between foot and insole, for increasing depth of the damaged tissue from the skin surface.

**Figure 9:**
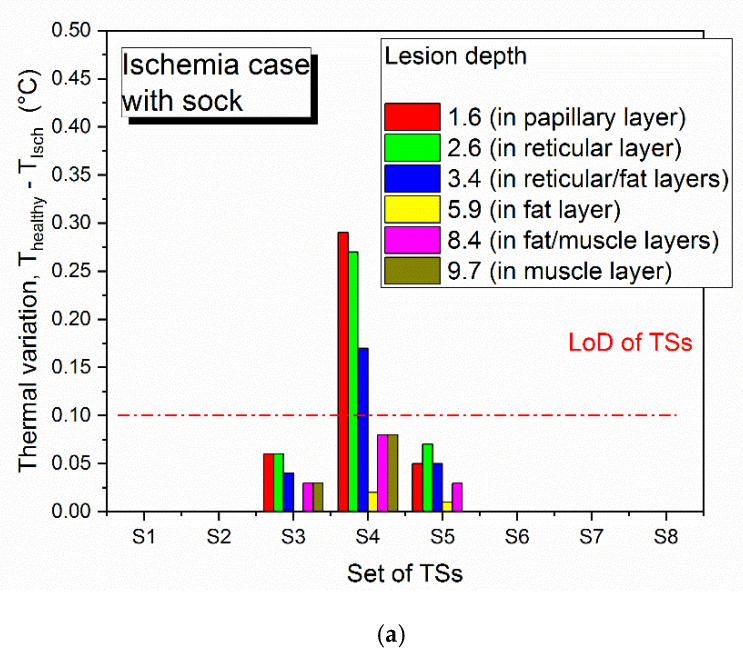

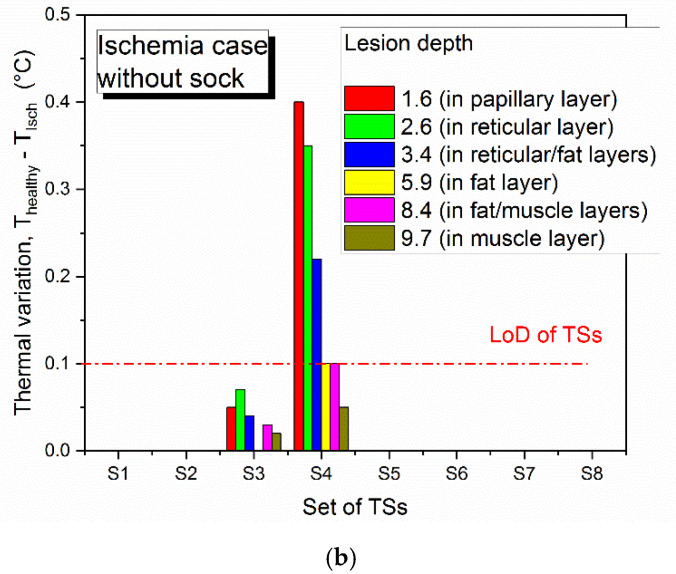
Limit of detection of thermal sensors (TSs) for subcutaneous ischemic lesion located in proximity of S4, (**a**) with sock and (**b**) without sock between foot and insole, for increasing depth of the damaged tissue from the skin surface.

Figure 7 shows the temperature distribution through the foot-insole system, for a 2.6 mm deep inflammation located nearby S4 sensor (see Figure 2). Figure 7a depicts a top view of the 3D foot-insole model: a uniform temperature of 36.4 °C can be found into the muscle layer of the foot, whereas the insole surface in contact with the foot is colder (about 24 °C). As can be seen from the plane view of the foot plantar (Figure 7b), the subcutaneous inflammation heats locally the above tissue, and this effect is also assessed on the insole surface (Figure 7c). The lesion was placed nearby one of the eight temperature sensors that monitor the main foot points where impending subcutaneous tissue damage are likely to occur. Figure 8 and Figure 9 show the variation of temperature, with respect to the value measured to healthy tissue (T_healthy_), measured by each sensor in case of inflammation (T_infl_) and ischemia (T_isch_), respectively, for increasing lesion depth within the foot tissue, with and without the thermal resistance related to the presence of a sock between foot and insole. In case of inflammation, the temperature variation is calculated as (T_infl_ − T_healthy_), for ischemia as (T_isch_ − T_healthy_). As reported in Figure 8 and Figure 9, is it clearly highlighted in what conditions the eight TSs are able to point out both a potential at-risk condition and the sensor closest to the damaged tissue. For the inflammation case (Figure 8), the system is able to detect a lesion up to the muscle layer depth, both in presence and absence of sock between foot and insole. The closest sensor (S4) measures the higher thermal variation with respect to the remaining sensors. Of these, only the two sensors near S4, S3 and S5, measure a meaningful thermal variation. The presence of the sock between foot and insole does not affect the detection capability, only a slight decrease of the temperature variation (T_infl_ − T_healthy_) is assessed. For the ischemia case, the resolution is limited to reticular/fat layer, being the temperature variation due to the lesion lower than inflammation. In addition, in this case, the presence of the sock slightly affects the heat transfer from the foot to the TSs. The detection capability of subcutaneous at-risk lesion is related to the limit of detection of the temperature sensors (0.1 °C for Maxim MAX30205): based on this assumption, FEA results demonstrated the successful detection of subcutaneous inflamed lesions located up to the muscle layer, and ischemic damage located not below the reticular/fat layer.

### 3.2. Characterization of Custom Pressure and Glucose Sensors.

As previously reported, an attempt to fabricate and characterize pressure and glucose sensors has been tried with the aim to realize a second prototype based on a fully custom smart insole with enhanced sensing functionalities. In the following, preliminary results about the response of microfabricated pressure and glucose sensors characterized when operating in real conditions were presented.

#### 3.2.1. Mechanical Properties of Piezoelectric Pressure Sensors

**Figure 10:**
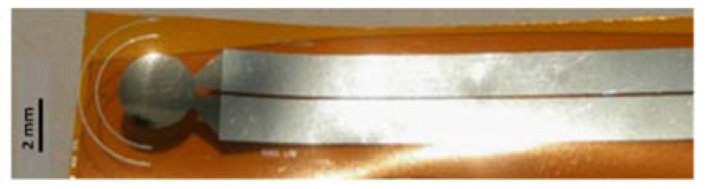
Photo of the fabricated device.

Figure 10 shows a photograph of the final microfabricated pressure sensor.

**Figure 11:**
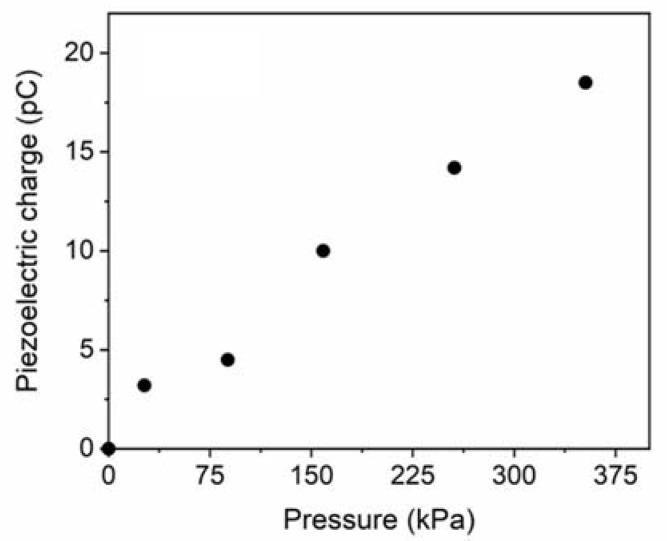
Piezoelectric charge generation from our pressure sensors for increasing applied load.

Mechanical properties of AlN sample were investigated by nanoindentation tests, obtaining an average value of Young modulus equal to 293 ± 15 GPa. The dielectric constant of AlN film was determined by CV measurements, obtaining a value of ε_r_ = 10.55 ± 0.25. The experimental custom set-up previously described was used to evaluate the electric charge generation of the realized sensor, when it is deformed by an applied external mechanical stress. A periodic vertical force was applied on the top of the device surface at a sampling frequency of 5 Hz, in the range 0–4 N, corresponding to a pressure range of 0–358 kPa that is coherent with the pressure variation detectable on the plantar foot. Figure 11 shows how the electric charge generated by the sensor increases proportionally with the applied pressure. From the average slope of the fitting straight line, the AlN piezoelectric coefficient d33 was determined be 4.5 ± 0.3 pC/N. Considering the experimental piezoelectric constant d_31_ = −0.7 ± 0.1 C/m^2^, the Figure of Merit for our full custom pressure sensors is calculated be 4.7 ± 1 GPa, using the formula $d_{31}^{2}/\left(\varepsilon_{0}\varepsilon_{r}\right)$ [76].

This value is lower than FOM values reported in literature for AlN piezoelectric devices, however the comparison is difficult because it is obtained considering devices realized on silicon [77]. The results, mainly in terms of piezo-generation response in the mechanical pressures range of interest, encouraged the potential integration of the fabricated device into a future prototype of engineered insole for monitoring the feet pressures of diabetic patients. The small size and geometry could permit their positioning on different insole points to collect pressure measurements coming from different sites, obtaining more accurate tests, and maximizing the effectiveness of the system. Moreover, piezoelectric transduction method could allow for insole prototypes working in a self-powering way, answering to the urgent demand of design and solutions for self-powered or ultra-low power consumption devices.

#### 3.2.2. Detection of Glucose: Experimental Linear Sensing Range Identification and Limit of Detection (LOD)

Different electrochemical methods were applied for the characterization and the quantitative detection of glucose in a phosphate buffer solution (PBS), which mimics the sweat medium. Amperometric and voltammetric characterizations were carried out in a PBS ferrocenemethanol solution as a redox reference system (Figure 4) and different concentrations of glucose were added to redox probe solution in order to evaluate the performance of the electrodes in terms of linear sensing range, limit of detection and sensitivity. In order to better evaluate a glucose biosensor performance, these metrics as well as response time, and stability should be evaluated depending on the application and on the working environment of the sensor [78]. A blood glucose sensor, for example, should be selective, rapid, and reliable, requirements that impact to sensitivity, response time and stability, respectively [79]. However, considering the sweat-based sensing systems, the ideal biosensor should primarily exhibit a very high selectivity towards changes in glucose concentration in the presence of competing and noncompeting analytes found in the sweat [74]. Moreover, to predict the correct concentration of glucose, in our case the sensor response should be in the linear response output range [80,81].

As reported in the figures below, authors evaluated the linear sensing range and detection parameters of the fabricated sensors applying chronoamperometry (CA) and cyclic voltammetry (CV) methods (Table 4).

**Figure 12:**
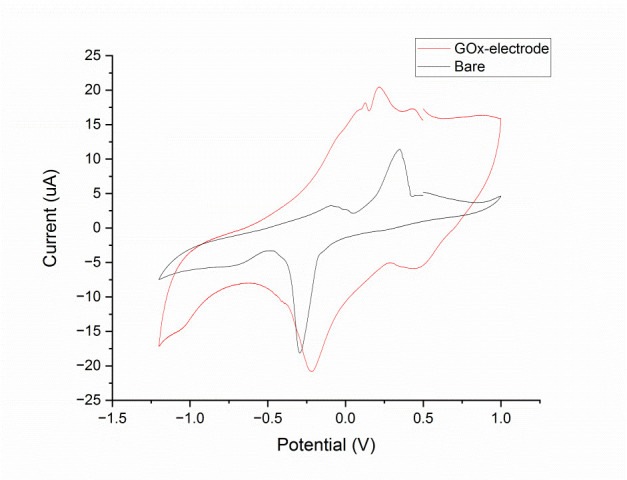
Comparison between bare and GOx enzyme functionalized electrode in a PBS/ferrocenemethanol solution as a redox reference system.

Figure 12 reports the experimental results on cyclic voltammetry spectra of bare and GOx functionalized electrodes. It can be noted that, when the electrode was covered by GOx enzyme, the current peak increased considerably regarding the bare electrode. In addition, the oxidation and reduction potential peaks of the ferrocenemethanol were shown at ±0.3 V.

**Figure 13:**
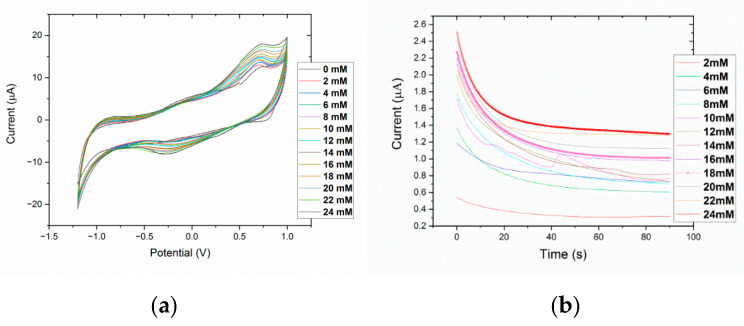
Cyclic Voltammetric (**a**) and Chronoamperometric analysis (**b**) to detect a linear sensing range for GOx-immobilized electrode.

Then, we perform an experimental assessment of the linear sensing detection range of the sensors, applying chronoamperometry (CA) and cyclic voltammetry (CV) with different D-glucose concentrations in the PBS/redox probe solution described above (Figure 13).

**Figure 14:**
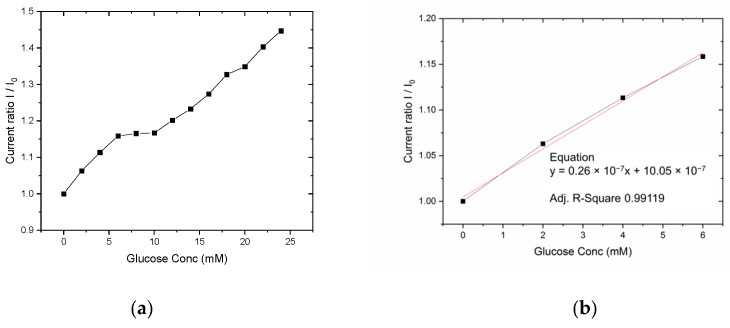
Sensor response as normalized current peak (+0.72 V) to different glucose concentrations in PBS: 0.1 mmol/L ferrocenemethanol (**a**) and linear fit for experimental data range 0–6 mM (**b**).

A higher current and linear response was obtained in voltammetric analysis, reporting a linear sensing range up to 6 mM and from 10 mM to 24 mM (Figure 14). In our application related to sweat glucose detection, the lower concentration range up to 6 mM is required for tests, considering that typical glucose concentrations in sweat are in the range 5–100 µM. The cyclic voltammetry data reported in Figure 13a, show a clear behavior of the current peaks at +0.72 V, which peak values increase with glucose concentrations. The same proportional correlation is reported by the Figure 13b with a chronoamperometric analysis; considering the higher current values obtained by the CV technique, and evaluating the future integration of the investigated sensor into the proposed monitoring insole, the methods with an higher current level are preferred for a better analog-to-digital (A/D) front-end reliability, keeping a low cost of the final product. To this aim the sensor response up to 24 mM has been investigated with CV (Figure 14a) and a linear fit of the experimental data up to 6 mM is reported the Figure 14b.

The sensor exhibits a linear behavior in the lower concentration range with a small plateau between 6 and 10 mM; the lower concentrations range (0–6 mM) has been chosen as the final application range because of the expected glucose dilution between serum and sweat (about one order of magnitude) [82].

**Figure 15:**
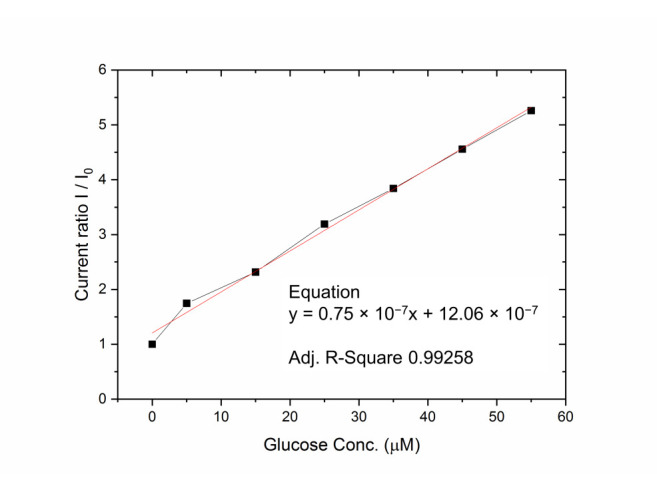
Sensor response as normalized current peak (+0.72 V) to low range glucose concentrations in PBS: 0.1 mmol/L ferrocenemethanol, and linear fit for experimental data range 0–60 µM.

Then, using the same electrochemical method, the LoD is calculated from experimental normalized responses in the low-range glucose concentrations (0–60 µM, see Figure 15) from the formula defined by the IUPAC [83]. The chosen concentration range was 0–60 µM, a typical and operative range for effective identification of glucose levels abnormalities in sweat [81].

The disposable glucose sensors exhibit a linear response for both 0–6 mM (0–108 mg/dL) and 0–60 µM ranges, with a detection limit of 3.27 µM (0.058 mg/dL) and a sensitivity of 21 µA/mM cm^2^ in the testing solution. Future investigations will be related to the membrane encapsulation of the enzymatic layer in order to better control the oxygen and glucose diffusion/reaction and to enhance the sensor reliability.

## 4. Conclusions

A smart multisensory hardware-software monitoring platform for diabetic patients was presented, in which an antibacterial polyurethane-based insole was proposed as a reliable platform for integration of an array of minimally invasive and low power commercial and proprietary sensors distributed on the plantar surface. The effectiveness of the temperature sensor array to detect early signs of subcutaneous inflamed or ischemic lesions placed at increasing depth from the foot skin was evaluated by FEA simulations performed on a 3D model of insole-foot assembly. The microfabrication processes and preliminary results of functional characterization of flexible AlN based piezoelectric pressure sensors and glucose sensors were presented. The figure of merit for the full custom pressure sensors is 4.7 ± 1 GPa. The disposable glucose sensors exhibit a 0–6 mM (0–108 mg/dL) glucose concentration optimized linear response (for sweat-sensing), with a LOD of 3.27 µM (0.058 mg/dL) and a sensitivity of 21 µA/mM cm^2^ in the PBS solution. Experimental and numerical results allowed to assess the technical prerequisites and experimental sensing performance for their future integration into a second prototype based on a full custom smart insole with enhanced sensing functionalities.

## Figures and Tables

**Table 1:** Conditioning parameters of Cyclic Voltammetry for citrate buffer and sulfuric acid steps.

Conditioning Solution	0.1 M Citrate Buffer	0.05 M Sulfuric Acid
***Start E (V)***	0.5	0.5
***High E (V)***	1.7	1.0
***Low E (V)***	−1.2	−1.2
***Scans***	10	10
***Scan Rate (V s^−1^)***	0.1	0.1

**Table 2:** Thermophysical properties of insole material layers.

	Density (Kg/m^3^)	Thermal Conductivity (W/m/K)	Specific Heat (J/Kg/K)	Thickness (mm)
Kapton	1300	0.15	1100	0.36
Copper	8960	401	384	0.018
Polyurethane	374	0.06	1337	Upper: 1 Bottom: 3
Rubber	1100	0.13	2010	15

**Table 3:** Thermophysical properties of healthy and damaged tissue used in FEA simulations.

Tissue	Specific Heat (J/Kg/K)	Thermal Conductivity (W/m/K)	Density (Kg/m^3^)	Metabolic Heat Generation (W/m^3^)	Perfusion Rate * 10^−3^ (1/s)	Thickness (mm)
Epidermis	3589	0.235	1200	0	0	0.46
Papillary dermis	3300	0.445	1200	368.1	0.18	1.67
Reticular dermis	3300	0.445	1200	368.1	1.26	1.67
Fat	2674	0.185	1000	368.3	0.08	5
Muscle	3600	0.51	1085	684.2	2.7	25
Inflammation	2450	0.558	1037	5262.5	6.95	2.5
Ischemia	2450	0.1	1037	342.1	0.405	2.5

**Table 4:** Chronoamperometry and cyclic voltammetry parameters.

Parameters for Chronoamperometry	Set E (V)	+0.3
	Duration (s)	3
	Interval Time (s)	0.1
	N. of repeats	30
Parameters for Cyclic Voltammetry	Start E (V)	+0.5
	High E (V)	1.0
	Low E (V)	−1.2
	Scans	4
	Scan Rate (V s^−1^)	0.1

## Data Availability

Not applicable.

## References

[1] Apelqvist J., Elgzyri T., Larsson J., Löndahl M., Nyberg P., Thörne J. (2011). Factors related to outcome of neuroischemic/ischemic foot ulcer in diabetic patients. J. Vasc. Surg..

[2] Said G., Said G., Krarup C. (2013). Chapter 33—Diabetic neuropathy. Handbook of Clinical Neurology.

[3] Ducic I., Short K.W., Dellon A.L. (2004). Relationship Between Loss of Pedal Sensibility, Balance, and Falls in Patients with Peripheral Neuropathy. Ann. Plast. Surg..

[4] Goyal M., Reeves N.D., Rajbhandari S., Ahmad N., Wang C., Yap M.H. (2020). Recognition of ischaemia and infection in diabetic foot ulcers: Dataset and techniques. Comput. Biol. Med..

[5] Prompers L., Huijberts M., Apelqvist J., Jude E., Piaggesi A., Bakker K., Edmonds M., Holstein P., Jirkovska A., Mauricio D. (2007). High prevalence of ischaemia, infection and serious comorbidity in patients with diabetic foot disease in Europe. Baseline results from the Eurodiale study. Diabetologia.

[6] Frykberg R.G., Lavery L.A., Pham H., Harvey C., Harkless L., Veves A. (1998). Role of Neuropathy and High Foot Pressures in Diabetic Foot Ulceration. Diabetes Care.

[7] Fernando M.E., Crowther R.G., Pappas E., Lazzarini P.A., Cunningham M., Sangla K.S., Buttner P., Golledge J. (2014). Plantar Pressure in Diabetic Peripheral Neuropathy Patients with Active Foot Ulceration, Previous Ulceration and No History of Ulceration: A Meta-Analysis of Observational Studies. PLoS ONE.

[8] Pataky Z., Golay A., Bounameaux H., Bobbioni-Harsch E., Assal J.P. (2003). Relationship between peripheral vascular disease and high plantar pressures in diabetic neuro-ischaemic patients. Diabetes Metab..

[9] Pataky Z., Assal J.-P., Conne P., Vuagnat H., Golay A. (2005). Plantar pressure distribution in Type 2 diabetic patients without peripheral neuropathy and peripheral vascular disease. Diabet. Med..

[10] Sutkowska E., Sutkowski K., Sokołowski M., Franek E., Dragan S. (2019). Distribution of the Highest Plantar Pressure Regions in Patients with Diabetes and Its Association with Peripheral Neuropathy, Gender, Age, and BMI: One Centre Study. J. Diabetes Res..

[11] Pitei D.L., Lord M., Foster A., Wilson S., Watkins P.J., Edmonds M.E. (1999). Plantar pressures are elevated in the neuroischemic and the neuropathic diabetic foot. Diabetes Care.

[12] Fernando M.E., Crowther R.G., Wearing S., Müller B., Wolf S. (2016). The Importance of Foot Pressure in Diabetes. Handbook of Human Motion.

[13] Fernando M.E., Crowther R.G., Lazzarini P.A., Sangla K.S., Wearing S., Buttner P., Golledge J. (2016). Plantar pressures are higher in cases with diabetic foot ulcers compared to controls despite a longer stance phase duration. BMC Endocr. Disord..

[14] Volmer-Thole M., Lobmann R. (2016). Neuropathy and Diabetic Foot Syndrome. Int. J. Mol. Sci..

[15] Perkins B.A. (2020). Rethinking Neuropathy in Type 1 Diabetes: Had We Lost Sight of What Matters Most?. Diabetes Care.

[16] Adams O.P., Herbert J.R., Howitt C., Unwin N. (2019). The prevalence of peripheral neuropathy severe enough to cause a loss of protective sensation in a population-based sample of people with known and newly detected diabetes in Barbados: A cross-sectional study. Diabet. Med..

[17] Chatwin K.E., Abbott C.A., Boulton A.J., Bowling F.L., Reeves N.D. (2020). The role of foot pressure measurement in the prediction and prevention of diabetic foot ulceration—A comprehensive review. Diabetes/Metab. Res. Rev..

[18] Anjos D.M.C., Gomes L.P.O., Sampaio L.M.M., Correa J.C.F., Oliveira C.S. (2010). Assessment of plantar pressure and balance in patients with diabet. Arch. Med. Sci..

[19] Drăgulinescu A., Drăgulinescu A.-M., Zincă G., Bucur D., Feieș V., Neagu D.-M. (2020). Smart Socks and In-Shoe Systems: State-of-the-Art for Two Popular Technologies for Foot Motion Analysis, Sports, and Medical Applications. Sensors.

[20] Pai S., Vawter P.T., LeDoux W.R. (2013). The Effect of Prior Compression Tests on the Plantar Soft Tissue Compressive and Shear Properties. J. Biomech. Eng..

[21] Niroomandi S., Perrier A., Bucki M., Payan Y. (2020). Real-time computer modeling in prevention of foot pressure ulcer using patient-specific finite element model and model order reduction techniques. Innov. Emerg. Technol. Wound Care.

[22] De Berardinis J., Trabia M., Dufek J.S. (2016). Review of Foot Plantar Pressure—Focus on the Development of Foot Ulcerations. Open Access J. Sci. Technol..

[23] Malhotra S., Bello E., Kominsky S. (2012). Diabetic Foot Ulcerations: Biomechanics, Charcot Foot, and Total Contact Cast. Semin. Vasc. Surg..

[24] Taborri J., Rossi S., Palermo E., Patanè F., Cappa P. (2014). A Novel HMM Distributed Classifier for the Detection of Gait Phases by Means of a Wearable Inertial Sensor Network. Sensors.

[25] Benbakhti A.S., Boukhenous S., Zizoua C., Attari M. An instrumented shoe for ambulatory prevention of diabetic foot ulceration. Proceedings of the 2014 4th International Conference on Wireless Mobile Communication and Healthcare—Transforming Healthcare Through Innovations in Mobile and Wireless Technologies (MOBIHEALTH).

[26] Yong K.F., Forero J.P., Foong S., Nanayakkara S. AH’15. Proceedings of the 6th Augmented Human International Conference, Marina Bay Sands.

[27] Tee K.S., Javahar Y.S.H., Saim H., Zakaria W.N.W., Khialdin S.B.M., Isa H., Awad M.I., Soon C.F. (2017). A Portable Insole Pressure Mapping System. TELKOMNIKA Telecommun. Comput. Electron. Control..

[28] Schneider W.L., Severn M. (2016). Prevention of Plantar Ulcers in People with Diabetic Peripheral Neuropathy Using Pressure-Sensing Shoe Insoles. 2017 Jun 1. CADTH Issues in Emerging Health Technologies.

[29] Aqueveque P., Osorio R., Pastene F., Saavedra F., Pino E. Capacitive Sensors Array for Plantar Pressure Measurement Insole fabricated with Flexible PCB. Proceedings of the 2018 40th Annual International Conference of the IEEE Engineering in Medicine and Biology Society (EMBC).

[30] Pappas I.P.I., Keller T., Mangold S., Popovic M.R., Dietz V., Morari M. (2004). A reliable gyroscope-based gait-phase de-tection sensor embedded in a shoe insole. IEEE Sens. J..

[31] Interlink Electronics Inc Force Sensing Resistor® Integration Guide and Evaluation Parts Catalog. https://www.sparkfun.com/datasheets/Sensors/Pressure/fsrguide.pdf.

[32] Razak A.H.A., Zayegh A., Begg R.K., Wahab Y. (2012). Foot Plantar Pressure Measurement System: A Review. Sensors.

[33] Motha L., Kim J., Kim W.S. (2015). Instrumented rubber insole for plantar pressure sensing. Org. Electron..

[34] Park J., Kim M., Hong I., Kim T., Lee E., Kim E.-A., Ryu J.-K., Jo Y., Koo J., Han S. (2019). Foot Plantar Pressure Measurement System Using Highly Sensitive Crack-Based Sensor. Sensors.

[35] Armstrong D.G., Holtz-Neiderer K., Wendel C., Mohler M.J., Kimbriel H.R., Lavery L.A. (2007). Skin Temperature Monitoring Reduces the Risk for Diabetic Foot Ulceration in High-risk Patients. Am. J. Med..

[36] Ring E.F.J., Ammer K. (2012). Infrared thermal imaging in medicine. Physiol. Meas..

[37] Etehadtavakol M., Ng E.Y.K. (2017). Assessment of foot complications in diabetic patients using thermography: A review. Application of Infrared to Biomedical Sciences.

[38] Bagavathiappan S., Philip J., Jayakumar T., Raj B., Rao P.N.S., Varalakshmi M., Mohan V. (2010). Correlation between plantar foot temperature and diabetic neuropathy: A case study by using an infrared thermal imaging technique. J. Diabetes Sci. Technol..

[39] Lavery L.A., Higgins K.R., Lanctot D.R., Constantinides G.P., Zamorano R.G., Armstrong D.G., Athanasiou K.A., Agrawal C.M. (2004). Home monitoring of foot skin temperatures to prevent ulceration. Diabetes Care.

[40] Lavery L., Peters E., Armstrong D. (2008). What are the most effective interventions in preventing diabetic foot ul-cers?. Intern. Wound J..

[41] Brem H., Sheehan P., Rosenberg H.J., Schneider J.S., Boulton A.J.M. (2006). Evidence-Based Protocol for Diabetic Foot Ulcers. Plast. Reconstr. Surg..

[42] Ilo A., Romsi P., Mäkelä J. (2020). Infrared Thermography and Vascular Disorders in Diabetic Feet. J. Diabetes Sci. Technol..

[43] Peregrina-Barreto H., Morales-Hernandez L.A., Rangel-Magdaleno J.J., Avina-Cervantes J.G., Ramirez-Cortes J.M., Morales-Caporal R. (2014). Quantitative Estimation of Temperature Variations in Plantar Angiosomes: A Study Case for Diabetic Foot. Comput. Math. Methods Med..

[44] Martín-Vaquero J., Encinas A.H., Queiruga-Dios A., Bullón J.J., Martínez-Nova A., González J.T., Bullón-Carbajo C. (2019). Review on Wearables to Monitor Foot Temperature in Diabetic Patients. Sensors.

[45] American Diabetes Association (2009). Diagnosis and classification of diabetes mellitus. Diabetes Care.

[46] Röder P.V., Wu B., Liu Y., Han W. (2016). Pancreatic regulation of glucose homeostasis. Exp. Mol. Med..

[47] Papatheodorou K., Banach M., Bekiari E., Rizzo M., Edmonds M. (2018). Complications of Diabetes 2017. J. Diabetes Res..

[48] https://www.webmd.com/diabetes/guide/risks-complications-uncontrolled-diabetes.

[49] Lipska K.J., Ross J.S., Miao Y., Shah N.D., Lee S.J., Steinman M.A. (2015). Potential Overtreatment of Diabetes Mellitus in Older Adults with Tight Glycemic Control. JAMA Intern. Med..

[50] Brod M., Kongsø J.H., Lessard S., Christensen T.L. (2009). Psychological insulin resistance: Patient beliefs and implications for diabetes management. Qual. Life Res..

[51] Huang J., Zhang Y., Wu J. (2020). Review of non-invasive continuous glucose monitoring based on impedance spectroscopy. Sens. Actuators A Phys..

[52] Moyer J., Wilson D., Finkelshtein I., Wong B., Potts R. (2012). Correlation Between Sweat Glucose and Blood Glucose in Subjects with Diabetes. Diabetes Technol. Ther..

[53] Hauke A., Simmers P.C., Ojha Y.R., Cameron B.D., Ballweg R., Zhang T., Twine N.B., Brothers M., Gomez E., Heikenfeld J. (2018). Complete validation of a continuous and blood-correlated sweat biosensing device with integrated sweat stimulation. Lab Chip.

[54] Lee H., Hong Y.J., Baik S., Hyeon T., Kim D. (2018). Enzyme-Based Glucose Sensor: From Invasive to Wearable Device. Adv. Health Mater..

[55] Vashist S.K. (2012). Non-invasive glucose monitoring technology in diabetes management: A review. Anal. Chim. Acta.

[56] Kim J., Campbell A.S., Wang J. (2018). Wearable non-invasive epidermal glucose sensors: A review. Talanta.

[57] Hsu W.-C., Sugiarto T., Chen J.-W., Lin Y.-J. (2018). The Design and Application of Simplified Insole-Based Prototypes with Plantar Pressure Measurement for Fast Screening of Flat-Foot. Sensors.

[58] Coates J., Chipperfield A., Clough G. (2016). Wearable Multimodal Skin Sensing for the Diabetic Foot. Electronics.

[59] Tao J., Dong M., Li L., Wang C., Li J., Liu Y., Bao R., Pan C. (2020). Real-time pressure mapping smart insole system based on a controllable vertical pore dielectric layer. Microsyst. Nanoeng..

[60] Rescio G., Leone A., Francioso L., Losito P., Genco E., Crudele F., D’Alessandro L., Siciliano P. (2019). Fully Integrated Smart Insole for Diabetic Foot. Ambient Assisted Living.

[61] Ferber R., Webber T., Everett B., Groenland M. (2013). Validation of Plantar Pressure Measurements for a Novel in-Shoe Plantar Sensory Replacement Unit. J. Diabetes Sci. Technol..

[62] Signore M.A., Rescio G., De Pascali C., Iacovacci V., Dario P., Leone A., Quaranta F., Taurino A., Siciliano P., Francioso L. (2019). Fabrication and characterization of AlN-based flexible piezoelectric pressure sensor integrated into an implantable artificial pancreas. Sci. Rep..

[63] Guo W.C., Xu H., Gao X.Q., Hou X.L., Li Y. (2016). A Modified Method for Hardness Determination from Nanoindentation Experiments with Imperfect Indenters. Adv. Mater. Sci. Eng..

[64] (2008). Evaluation of Measurement Data—Guide to the Expression of Uncertainty in Measurement. Part 3.

[65] Khadka R., Aydemir N., Carraher C., Hamiaux C., Colbert D., Cheema J., Malmström J., Kralicek A., Travas-Sejdic J. (2019). An ultrasensitive electrochemical impedance-based biosensor using insect odorant recep-tors to detect odorants. Biosens. Bioelectron..

[66] Kuklane K., Afanasieva R., Burmistrova O., Bessonova N., Holmér I. (1999). Determination of Heat Loss from the Feet and Insulation of the Footwear. Int. J. Occup. Saf. Ergon..

[67] Hashan M., Hasan K.M.F., Khandaker F.R., Karmaker K., Deng Z., Zilani M.J. (2017). Functional Properties Improvement of Socks Items Using Different Types of Yarn. Int. J. Text. Sci..

[68] Pennes H.H. (1948). Analysis of Tissue and Arterial Blood Temperatures in the Resting Human Forearm. J. Appl. Physiol..

[69] Bhargava A., Chanmugam A., Herman C. (2014). Heat transfer model for deep tissue injury: A step towards an early thermographic diagnostic capability. Diagn. Pathol..

[70] Çetingül M.P., Herman C. (2010). A heat transfer model of skin tissue for the detection of lesions: Sensitivity analysis. Phys. Med. Biol..

[71] Cichowitz A., Pan W.R., Ashton M. (2009). The heel: Anatomy, blood supply, and the pathophysiology of pressure ulcers. Ann. Plast. Surg..

[72] Ruschkewitz Y., Gefen A. (2009). Cell-level temperature distributions in skeletal muscle post spinal cord injury as related to deep tissue injury. Med Biol. Eng. Comput..

[73] Aoi N., Yoshimura K., Kadono T., Nakagami G., Iizuka S., Higashino T., Araki J., Koshima I., Sanada H. (2009). Ultrasound Assessment of Deep Tissue Injury in Pressure Ulcers: Possible Prediction of Pressure Ulcer Progression. Plast. Reconstr. Surg..

[74] Herrman E.C., Knapp C.F., Donofrio J.C., Salcido R. (1999). Skin perfusion responses to surface pressure-induced ischemia: Implication for the developing pressure ulcer. J. Rehabil. Res. Dev..

[75] Linder-Ganz E., Gefen A. (2007). The Effects of Pressure and Shear on Capillary Closure in the Microstructure of Skeletal Muscles. Ann. Biomed. Eng..

[76] Roscow J.I., Pearce H., Khanbareh H., Kar-Narayan S., Bowen C.R. (2019). Modified energy harvesting figures of merit for stress- and strain-driven piezoelectric systems. Eur. Phys. J. Speéc. Top..

[77] Matloub R., Metzger T., Muralt P. (2013). Piezoelectric Al_1−x_Sc_x_N thin films: A semiconductor compatible solution for mechanical energy harvesting and sensors. Appl. Phys. Lett..

[78] Cargill A.A. (2016). Development of an Enzymatic Glucose Biosensor for Applications in Wearable Sweat-Based Sensing. Master’s Thesis.

[79] Oliver N.S., Toumazou C., Cass A.E.G., Johnston D.G. (2009). Glucose sensors: A review of current and emerging technology. Diabet. Med..

[80] Lavín Á., De Vicente J., Holgado M., Laguna M.F., Casquel R., Santamaría B., Maigler M.V., Hernández A.L., Ramírez Y. (2018). On the Determination of Uncertainty and Limit of Detection in Label-Free Biosensors. Sensors.

[81] Hwang D.-W., Lee S., Seo M., Chung T.D. (2018). Recent advances in electrochemical non-enzymatic glucose sensors—A review. Anal. Chim. Acta.

[82] Heikenfeld J. (2016). Non-invasive Analyte Access and Sensing through Eccrine Sweat: Challenges and Outlook circa 2016. Electroanalysis.

[83] IUPAC (1997). Compendium of Chemical Terminology.

